# Analysis of second opinion programs provided by German statutory and private health insurance – a survey of statutory and private health insurers

**DOI:** 10.1186/s12913-021-06207-8

**Published:** 2021-03-09

**Authors:** Nadja Könsgen, Barbara Prediger, Ana-Mihaela Bora, Angelina Glatt, Simone Hess, Victoria Weißflog, Dawid Pieper

**Affiliations:** 1 Institute for Research in Operative Medicine, Witten/Herdecke University, Cologne, Germany; 2 Medexo GmbH - Medical experts online, Berlin, Germany

**Keywords:** Second opinion, Second opinion programs, Health insurance, Informed decision making, Patient autonomy, Survey

## Abstract

**Background:**

Second medical opinions can give patients confidence when choosing among treatment options and help them understand their diagnosis. Health insurers in several countries, including Germany, offer formal second opinion programs (SecOPs). We systematically collected and analyzed information on German health insurers’ approach to SecOPs, how the SecOPs are structured, and to what extent they are evaluated.

**Methods:**

In April 2019, we sent a questionnaire by post to all German statutory (*n* = 109) and private health insurers (*n* = 52). In September 2019, we contacted the nonresponders by email. The results were analyzed descriptively. They are presented overall and grouped by type of insurance (statutory/private health insurer).

**Results:**

Thirty one of One hundred sixty one health insurers (response rate 19%) agreed to participate. The participating insurers covered approximately 40% of the statutory and 34% of the private health insured people. A total of 44 SecOPs were identified with a median of 1 SecOP (interquartile range (IQR) 1–2) offered by a health insurer. SecOPs were in place mainly for orthopedic (21/28 insurers with SecOPs; 75%) and oncologic indications (20/28; 71%). Indications were chosen principally based on their potential impact on a patient (22/28; 79%). The key qualification criterion for second opinion providers was their expertise (30/44 SecOPs; 68%).

Second opinions were usually provided based on submitted documents only (21/44; 48%) or on direct contact between a patient and a doctor (20/44; 45%). They were delivered after a median of 9 days (IQR 5–15). A median of 31 (IQR 7–85) insured persons per year used SecOPs. Only 12 of 44 SecOPs were confirmed to have conducted a formal evaluation process (27%) or, if not, plan such a process in the future (10/22; 45%).

**Conclusion:**

Health insurers’ SecOPs focus on orthopedic and oncologic indications and are based on submitted documents or on direct patient-physician contact. The formal evaluation of SecOPs needs to be expanded and the results should be published. This can allow the evaluation of the impact of SecOPs on insured persons’ health status and satisfaction, as well as on the number of interventions performed. Our results should be interpreted with caution due to the low participation rate.

**Supplementary Information:**

The online version contains supplementary material available at 10.1186/s12913-021-06207-8.

## Introduction

Second medical opinions serve to validate diagnosis and treatment decisions [1, 2]. Second opinions can help patients to understand their diagnoses and make them more confident when weighing treatment options [3, 4]. They have been demonstrated to reduce the number of surgeries [2, 5–8]. Therefore, second opinion programs (SecOPs) might also lead to cost savings. The first SecOP was introduced for elective surgeries in the USA in the 1970s [5]. A systematic review found that many studies on SecOPs for elective surgeries were published in the 1970s and 1980s and that most were conducted in the USA [9]. Since then, SecOPs have been assessed outside the USA, for example, in Switzerland [10] and Latin America [2]. Second opinions are now offered in other countries, such as Israel [11] and the Netherlands [12]. A survey of the general population in Germany found that 24% of the respondents had obtained one or more second opinion(s) in the past [13].

In Germany, health insurance coverage is provided by statutory health insurers (SHIs) and private health insurers (PHIs) (covering 85 and 11% of the population, respectively, in 2012). For most Germans, it is mandatory that they are insured by an SHI. The self-employed and employees with a gross annual income above a certain threshold (62,550€ in 2020) may choose a PHI instead of an SHI. For some groups, such as civil servants, it is mandatory that they are insured by a PHI.

The Social Code Book (SGB) provides the legislative framework for statutory health insurance. Within this framework, many details are delegated to self-regulated organizations of payers and providers, including the Federal Joint Committee (G-BA). The G-BA is responsible for issuing directives that define the coverage of benefits [14]. Accordingly, within the framework of §27b SGB V, the G-BA issued the Second Opinion Directive specifying eligible elective procedure types. The Second Opinion Directive sets standards, for example, regarding the provision of the second opinion and the qualification of the second opinion provider.

In 2019, the right of people with SHI coverage to obtain an independent second opinion according to the Second Opinion Directive was implemented for hysterectomies, tonsillectomies, and tonsillotomies. Shoulder arthroscopy and total knee arthroplasty were added in 2020 [15, 16], and an extension to amputations of the lower extremities in patients with diabetic foot syndrome [17] is planned. Further indications will be added in the future [18]. SHIs are free to continue offering additional SecOPs beyond the above indications and frequently do so.

Prior to the implementation of the Second Opinion Directive, we reviewed the SecOPs offered by SHIs based on the information provided on their websites. At that time, 50 % of SHIs provided at least one SecOP. The most common indication was orthopedics [19].

Following the recent implementation of the Second Opinion Directive, we decided to reassess the landscape of SecOPs in more depth by surveying health insurers directly. Because some PHIs also voluntarily provide SecOPs, we included both, SecOPs offered by PHIs and SHIs, to provide a full picture.

The aim of our survey was to collect and categorize information on SecOPs in Germany, including the characteristics, quality assurance aspects, formal evaluation processes, participation rates, and time to delivery of the SecOPs offered by SHIs and PHIs.

## Methods

We followed the guidelines on conducting and reporting of survey research by Kelley et al. [20].

This work is part of the ongoing ‘ZWEIT’ project on second opinions in Germany funded by the ‘Innovation Fund’ of the G-BA. The project is conducted in collaboration with Medexo, a German second opinion provider; the SHI AOK Nordost; the Brandenburg Medical School; and the Association of Statutory Health Insurance Physicians of Brandenburg.

We developed a questionnaire to identify the characteristics, quality assurance aspects, formal evaluation processes, participation rates, and time to delivery of the SecOPs offered by SHIs and PHIs. In some of our questions, we followed the categorization used in our previous review of SecOPs [19]. Furthermore, we integrated different aspects of the Second Opinion Directive into our questions on the qualification criteria of the second opinion provider and the provisions to ensure the independence of financial interests. The questionnaire was developed in several interdisciplinary meetings between researchers and collaborating experts from a second opinion provider.

The questionnaire was divided into 3 parts: The first part with general questions on SecOPs included 7 items and had to be filled in once. The questionnaire mainly targeted health insurers offering SecOPs. Some questions could be answered by health insurers without a SecOP (such as the question on whether the introduction of a SecOP was planned). One item included nominating a contact person for further questions/interviews. Accordingly, most questionnaires were not anonymized (except when no contact person or signed cover letter was provided). Part two (with 14 items about details on the SecOPs provided) and three (with 12 items about evaluations of the provided SecOPs) were addressed specifically to health insurers offering SecOPs because they directly referred to the SecOPs provided. Since some health insurers provide more than one SecOP, part two and three had to be filled in once for each SecOP. The questionnaire included a mixture of closed-ended and open-ended questions. The closed-ended questions included dichotomous questions or questions with a set of possible predefined answers (multiple answers were possible in some cases). Since we sent our survey to all SHIs and PHIs, we were unable to pilot test the questionnaire with some health insurers beforehand. Instead, pilot testing was performed by a second opinion provider who regularly cooperates with health insurers.

We identified the SHIs and PHIs via the homepages of statutory and private health insurance associations, respectively [21, 22]. In April 2019, we sent the questionnaire by post to all SHIs (*n* = 109) and PHIs (*n* = 52). Because the people responsible for SecOPs at the different health insurers were hard to identify, we sent the questionnaires to the central postal address and asked for the questionnaire to be referred to the responsible parties. The cover letter introduced our project on second opinions funded by the G-BA, including the collaborators. Furthermore, the aims and methodology of our study were described. Because health insurers were asked to complete the questionnaire and send it back, the consent of health insurers was implicitly provided, obviating the need to obtain separate informed consent. Health insurers who had neither participated in our study nor declined to participate were contacted again in September 2019, this time via e-mail, to increase the likelihood of a response. Due to the nature of the project, we could not perform nonresponder analysis.

One person extracted the questionnaire data (AB or AG) into an Excel spreadsheet developed a priori, and another person checked the accuracy of the extracted data (NK). Discrepancies were discussed until a consensus was achieved. We analyzed the results descriptively and presented them overall and grouped by type of insurer (private/statutory). The health insurers’ approach to SecOPs was analyzed based on the number of health insurers, while the structure and evaluation of SecOPs were analyzed based on the number of SecOPs (because a health insurer may provide several SecOPs).

## Results

We initially contacted 109 SHIs and 52 PHIs and recontacted 84 SHIs and 41 PHIs. Overall, 14 health insurers refused to participate (8 PHIs and 7 SHIs). Their reasons included that the insurer offered no SecOP, lacked resources, and a second opinion provider participated in the main ‘ZWEIT’ project. Overall, 31 health insurers (6 private, 24 statutory, and 1 anonymous health insurer) participated in our survey, leading to an overall response rate of 19%. The overall results included all types of health insurers (SHIs, PHIs, and the anonymous health insurer).

**Fig. 1:**
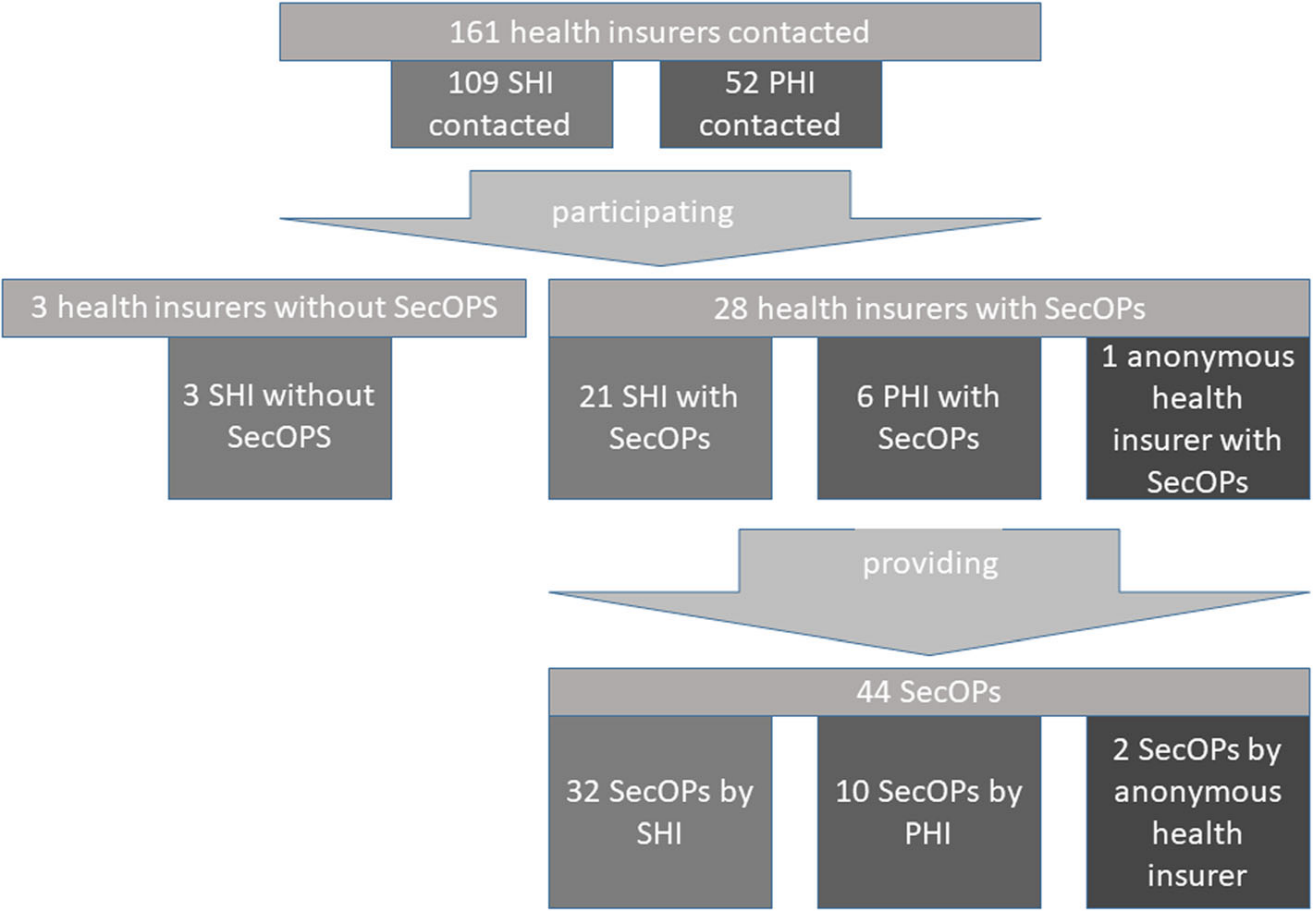
Overview on health insurers and SecOPs included

Approximately 90% of the participating health insurers (28/31) offered at least one SecOP. The median per health insurer was 1 SecOP (interquartile range (IQR) 1–2). Figure 1 shows the details on the number of SecOPs provided.

### Health insurers’ approach to SecOPs

Mostly, health insurers offered SecOPs for orthopedic (75%, 21/28) and oncologic indications (71%, 20/28). However, 21% (6/28) indicated that the type of indication was not restricted. More details on the included indications can be found in Table 1.

**Table 1:** Main results

Item	Results
Indications for which SecOPs are provided (%, n/number of health insurers with SecOPs)^a^	Orthopedics (75%, 21/28) Oncology (71%, 20/28) Cardiology (11%, 3/28) Gynecology (11%, 3/28) Pediatrics (11%, 3/28) Urology (11%, 3/28) Dermatology (7%, 2/28) Ear, nose, and throat (7%, 2/28) Gastroenterology (7%, 2/28) General and visceral surgery (7%, 2/28) Ophthalmology (7%, 2/28) Other (18%, 5/28) No restriction (21%, 6/28)
Method of ensuring independence of financial interests (%, n/number of SecOPs)^a^	Second opinion providers prohibited from - conducting the subsequent procedure (66%, 29/44) - working for the same company as the first opinion provider (57%, 25/44) Other (20%, 9/44)
Delivery route^c^ (%, n/number of SecOPs)^b^	Outsourcing to an external service provider (45%, 20/44) Contracts with selected health service provider (32%, 14/44) Other (9%, 4/44), e.g. specific university hospitals No (valid) answer (14%, 6/44)
Basis for second opinion (%; n/number of SecOPs)^b^	Submitted documents only (48%, 21/44) Direct contact between patient and doctor (45%, 20/44) Contact by phone (2%, 1/44) No (valid) answer (5%, 2/44)
Conduct of evaluation of SecOPs (past or currently) (%; n/number of SecOPs)^b^	Yes (27%, 12/44) No (50%, 22/44) No (valid) answer (23%, 10/44)
Conduct of evaluations in the following 2 years? (%, n/number of SecOPs without evaluations)^b^	Yes (45%, 10/22) No (45%, 10/22) No (valid) answer (9%, 2/22)
Insured persons obtaining SecOPs median per year, iqr (n SecOPs)	31, 7–85 (26)
Time to delivery of second opinion median, IQR (n SecOPs)	9 days, 5–15 (23)

^a^ multiple answers possible

^b^ multiple answers NOT possible

^c^ For details, see Fig. 4

*IQR* Interquartile range

**Fig. 2:**
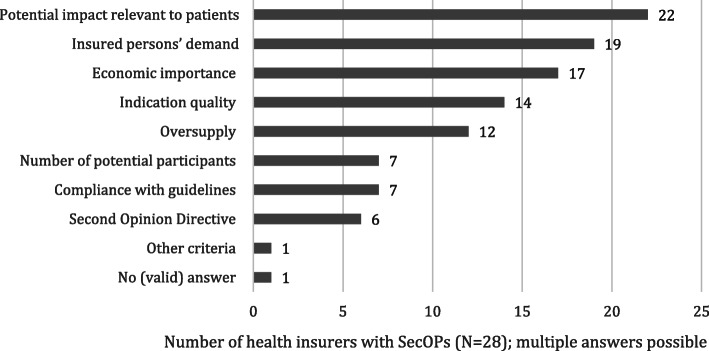
Selection criteria for the included indications

The criteria used by health insurers to select indications for SecOPs are presented by order of importance (Fig. 2, Additional file 1). The majority of health insurers with SecOPs (79%, 22/28) selected eligible indications based on the ‘potential impact relevant to patients’. This included conditions with serious impacts on the patients’ health status. Examples are burdening indications that restrict the patient’s (everyday) life or lead to high mortality. Another criterion was the ‘insured persons’ demand’ (68%, 19/28). For example, there might be indications for which patients requested a second opinion irrespective of the offered SecOPs more often. ‘Economic importance’ (61%, 17/28) includes the costs of (unnecessary) treatments/surgeries. ‘Indication quality’ refers to the possibility that there are some indications with a tendency of providing inadequate treatments. It partially overlaps with the criterion ‘oversupply’, which means that there might be surgeries that are too often performed in cases where a more conservative treatment, for example, would be a better option. The criterion ‘compliance with guidelines’ includes indications for which the health insurer denotes as having insufficient compliance with treatment guidelines. For further information on the health insurers’ approach to SecOPs, see Additional file 1.

### Structure of SecOPs

**Fig. 3:**
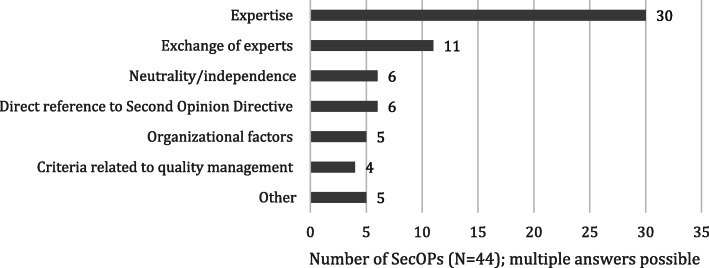
Qualification criteria for SecOP providers

One important step in the second opinion process is to define the qualification criteria for second opinion providers (see Fig. 3). For the majority of SecOPs (68%, 30/44), ‘expertise’ was considered (including extensive experience, training, specialization, position of chief physician or professor, scientific knowledge, and certification). ‘Exchange of experts’, for instance, was chosen when several physicians or medical professionals from other disciplines cooperated on the second opinion. ‘Neutrality/independence’ means that the second opinion should be provided independently from the first opinion provider (or other (financial) influencing factors). This was fulfilled either by labeling the second opinion provider as ‘independent’ or by providing a further explanation. Nonetheless, in another question, we assessed how independence was ensured. ‘Direct reference to the Second Opinion Directive’ was chosen whenever the health insurer directly referred to the qualification criteria stated in §27b SGB V. ‘Organizational factors’ included factors such as the time to delivery of the second opinion and the availability of physician appointments in a timely manner and at a nearby location. ‘Criteria related to quality management’ included results from patients’ surveys, quality reports, or other quality assurance procedures not further specified by the health insurers.

Mainly, independence of financial interests was ensured by prohibiting the second opinion provider from conducting the subsequent intervention (66%, 29/44) (see Table 1).

**Fig. 4:**
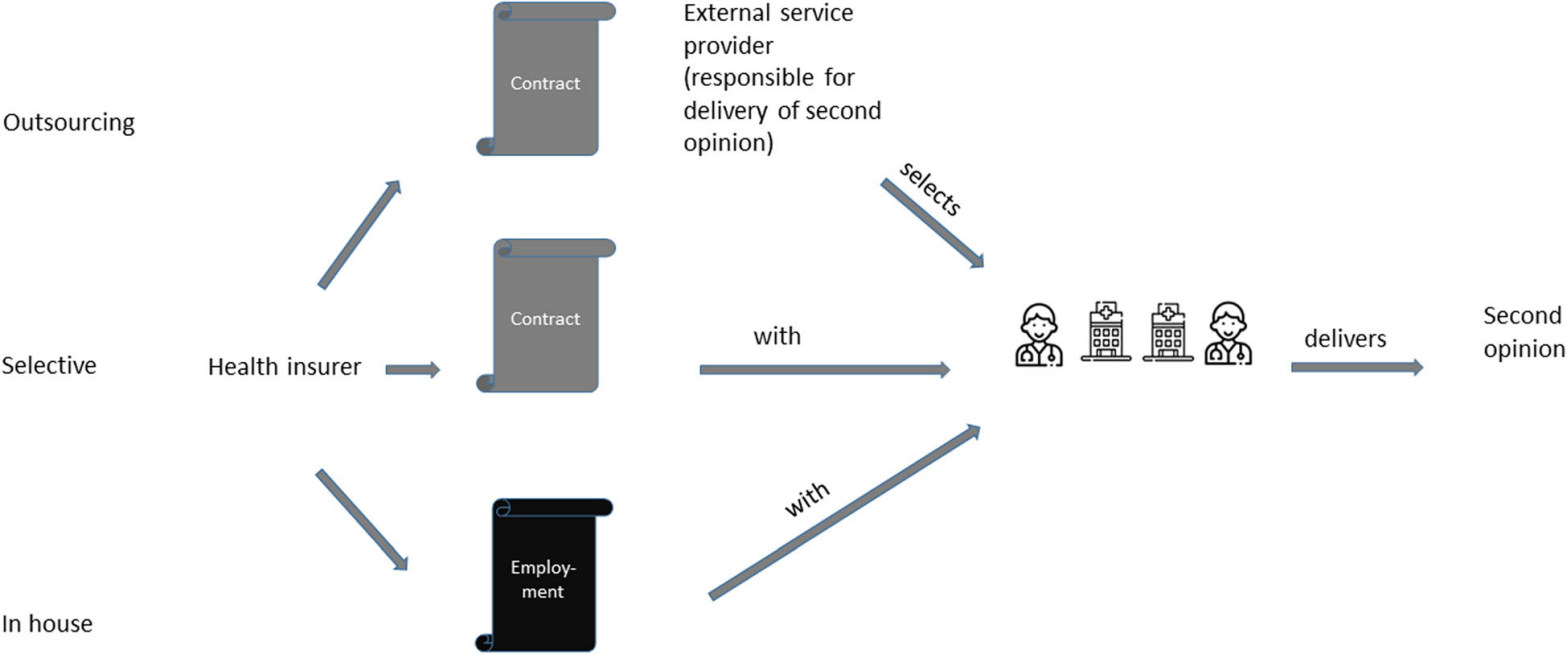
Explanation of different ways of second opinion provision. Icons made by Freepik and geotatah from www.flaticon.com

The differences between several methods of provision (outsourcing/selective/in house) are visualized in Fig. 4.

In 45% (20/44) of the SecOPs, the delivery of the second opinions was outsourced. In 32% (14/44) of the SecOPs, the second opinions were conducted by selected health service providers with established contracts with the health insurer (14/44). The basis of second opinions was dominated by two methods: 48% (21/44) of the SecOPs provided second opinions based on submitted documents only, and 45% (20/44) of the SecOPs provided second opinions based on direct contact between patients and doctors (see Table 1).

For approximately half of the SecOPs (52%, 23/44), timelines were agreed upon in the contracts between health insurers and second opinion providers; while for 45% (20/44) of the SecOPs, no such agreement was made. For the remaining 2% (1/44) of SecOPs no (valid) answer was given. Participation in SecOPs was free of charge to the insured person in 95% (42/44) of SecOPs. In the case of free-of-charge SecOPs, the health insurer directly paid the second opinion provider in 98% of the SecOPs (41/42). For the remaining 2% (1/42) of the SecOPs, the insured person had to make an advance payment. For further information about the structure of the SecOPs provided, see Additional file 2.

### Evaluations of SecOPs

A median of 31 insured persons (IQR 7–85, *N* = 26 values based on the means for the last available full-year dataset) participated in the SecOPs. The median age of the participants was 58 years (IQR 56–60, *N* = 23). Half were female (median 50%, IQR 40–54%, *N* = 25). The second opinions were delivered after a median of 9 days (IQR 5–15, N = 23). For further information on participants’ characteristics, see Additional file 3.

Program evaluations were stated to have been performed for only 27% (12/44) of the SecOPs. For nearly half of the SecOPs stating not to have performed evaluations, the conduct of evaluations was planned for the following 2 years (10/22; 45%), while another 45% did not plan to perform such evaluations (10/22). For the remaining 9% (2/22) no valid answer was provided (Table 1). The most important criteria measured during evaluations that have been performed were the satisfaction of the insured (9/12) and the agreement between first and second opinions (8/12). Furthermore, costs (7/12), second opinion quality (6/12), and other criteria (5/12) were measured. The median level of agreement between first and second opinions was 3 on a scale of 1–5 (smaller values indicating less agreement) when only taking into account the cases where evaluations were stated to have been performed and measurements of the agreement between the first and second opinions during evaluations were stated to have occurred. Otherwise, when taking into account all answers made regarding the level of agreement (some health insurers stated a level of agreement, even though conduct of evaluations or measurements of the level of agreement were stated not to have been performed), the median level of agreement was 2.

Evaluation reports existed for only 67% of the SecOPs that stated that evaluations have been performed (8/12). For 25% (3/12), no report existed, and 8% (1/12) have no (valid) answer. Health insurers were willing to make the report available for only 25% of the SecOPs with a report (2/8), they refused for 63% (5/8) to make the report available and did not provide a (valid) answer for 13% (1/8). For detailed information about evaluations, see Additional file 4.

### Results by type of insurer

SHIs provided a median of 1 SecOP (IQR 1–1), and PHIs provided a median of 2 SecOPs (IQR 1–2). Overall, the SecOPs for PHIs tended to be less restrictive with a wider range of fields. Fifty percent of PHIs (3/6) stated to have no restrictions (vs. 14% or 3/21).

The second opinion process also differed between SHIs and PHIs. For example, the variability in the qualification criteria for the second opinion provider was higher in the PHI subgroup.

Outsourcing was the most important way to deliver second opinions for the PHI subgroup (60% or 6/10 vs. 38% or 12/32 for SHIs). In contrast, many SHIs had established contracts with selected health care providers (44% or 14/32 vs. 0% or 0/10 for PHIs). The proportion of second opinions based on submitted documents only was larger for PHIs (60% or 6/10 vs. 41% or 13/32 for SHIs).

SecOPs have considerably more frequently been evaluated by PHIs (60% or 6/10 vs. 19% or 6/32 for SecOPs by SHIs). For further information on the results grouped by type of insurer, see Additional files 1-4.

## Discussion

German health insurers focus their SecOPs for orthopedic and oncologic indications. Second opinions based on submitted documents only and personally delivered second opinions were equally weighted in responses. It was shown, that only half of the SecOPs went through a formal evaluation process or, if not, planned such a process in the future. The number of participants in the SecOPs provided is relatively low.

The number of SecOPs that have been or will be evaluated is surprisingly low, particularly with regard to the fact that §140a SGB V, which applies to most of the SecOPs by SHIs, mandated that evaluations are performed at the time of the survey. However, this obligation has recently been repealed. It remains unclear whether those completing the survey were simply not aware that evaluations were or will be performed, whether the question was misunderstood, or whether parts of the SHIs did not comply with the obligation.

The relatively low number of insured persons who use SecOPs is surprising. Of course, we included a broad range of health insurers with a high variation in the number of insured persons (which leads to a wide range in the number of participants in the SecOPs provided). Nevertheless, most SecOPs seem to include only a few participants. Benbassat found that people with a lower socioeconomic status and lower education are less likely to seek a second opinion [23]. Additionally, young and educated persons are more likely to be informed by physicians about the possibility of seeking a second opinion [23]. It might be possible that many insured persons do not know about the SecOPs provided or that the information policy by health insurers only reaches a subset of insured persons.

Participants in the SecOPs provided were older than the mean age in Germany in 2019 [24] (58 years vs. 44.5 years). This agrees with the fact that older people tend to have higher utilization rates of health services [25]. In this context, the high proportion of second opinions provided based on submitted documents only (which usually refers to the electronic provision of documents) is very interesting since older people tend to use information and communication technologies less often than younger age groups [26]. Furthermore, most people prefer to receive their second opinions based on direct patient-physician contact [13]. The high share of second opinions based on documents only (as well as the focus on orthopedic and oncological indications) might at least partially be explained by the relatively high share of SecOPs provided via outsourcing. Both indications are focal areas for two important German second opinion providers that offer second opinions based on submitted documents and cooperate with several health insurers.

Furthermore, from a pragmatic point of view, once a process for forwarding the relevant documents from the patient to the physician is established, the organization of second opinions based on documents only is simple. After that, there is no need for patients to make an appointment, to travel to the physician or to sit in a physician’s waiting room. However, personal contact enables a physical examination that is necessary in some cases. Otherwise, when providing a SecOP with several indications or no restrictions regarding indications at all, it might not be possible to provide an appropriate physician that is within the patient’s reach for each indication. The aforementioned factors might explain why previous research showed high patient satisfaction for a SecOP offered by a second opinion provider who generally based second opinions on documents submitted online only (the mean patient age was comparable to our survey) [27]. As a result, patients may leave their reservations towards this manner of providing second opinions.

Another aspect important to patients’ needs is the timely provision of second opinions. Nearly half of the SecOPs had no contractual agreement on a timeline for providing second opinions. We found the mean time varied widely, which may, in part, be explained by the lack of a contractual agreement. Adding agreements on a timeline for providing second opinions to provider contracts might help to accelerate the second opinion process.

Analyses grouped by type of insurer suggests that there are differences in eligible indications, in the methods of providing second opinions, and in the existence of evaluation procedures between PHIs and SHIs. Because the number of PHIs included in the analysis was relatively low, single occurrences are given a higher weight for PHIs compared to SHIs. Another explanation for some differences is that the Second Opinion Directive directly applies only to SHIs. This is reflected by most of those results for which answers referred to the directive. Therefore, only SHIs named the Second Opinion Directive as a selection criterion for interventions/indications and directly referenced the qualification criteria stated in the Second Opinion Directive when asked about their qualification criteria for second opinion providers. However, the directive only played a minor role in both questions, even for the SHI subgroup. Independence of financial interests is also part of the Second Opinion Directive, and both SHIs and PHIs stated that they address this issue in their SecOPs, often by prohibiting second opinion providers from conducting the subsequent intervention. The large share of outsourcing second opinions for PHIs might play a role here because the two most important second opinion providers that cooperate with many health insurers prohibit their health care providers from conducting the subsequent intervention. The differences in the methods of providing second opinions might be explained by diverging market powers since SHIs insure considerably more people than PHIs, which enables them to contract with selected service providers. Our survey was performed about half a year after implementation of the Second Opinion Directive. Because of the short period between implementation of the directive and the performance of our survey (and the fact that most contracts for SecOPs might be made considerably prior to the implementation), it does not seem probable that the Second Opinion Directive has led to the introduction of the prohibition of the subsequent treatment. Second opinions for patients with indications included in the Second Opinion Directive must meet standards established by the Second Opinion Directive. Therefore, it does not seem reasonable to continue to offer individual SecOPs for these indications. When we conducted our survey, only two indications were included. Therefore, we assumed that the direct impact of the Second Opinion Directive on the SecOPs would be limited. The direct impact will become more interesting as the list of included indications expands. The expansion of second opinions according to the Second Opinion Directive (and the reduction in SecOPs that is likely to entail) will probably make the second opinion provision more homogenous. It remains unclear whether these changes will result in an improvement in health care. However, evaluation of the Second Opinion Directive in terms of utilization of second opinions, changes in the procedures performed, and impact of informed decision-making will begin soon.

### Comparison with other research

Our results are mostly in line with previous research by Pieper et al., which collected information on 59 SHIs providing 78 SecOPs from German SHIs’ websites in 2016 [19]. We covered 41% of the number of SHI with SecOPs and 41% of the number of SecOPs offered by SHIs that were identified by them. Our results agree that participating in SecOPs was mostly free of charge to the insured person, but the share of free-of-charge SecOPs identified in our present study for SHIs was higher (94% vs. 73%). There are some differences regarding the basis for providing second opinions, which have to be interpreted in the context that we did not allow multiple answers while they did. We tended to have lower shares of second opinions based on documents only (41% for SHIs vs. 63%) and of second opinions provided via phone (3% for SHIs vs. 14%). They stated that SecOPs provided by health insurer staff were delivered via phone. This might explain why we did not find any SecOPs that were provided by SHI staff while they found that 11% of second opinions were provided in house. Since they conducted their analysis before the implementation of the Second Opinion Directive, it would be possible to observe considerable effects of the directive on the SecOPs provided, such as the abolishment of the SecOPs provided. Notwithstanding, we did not observe a considerable effect on the number of SecOPs (since the median number of SecOPs offered by SHIs was the same in their analysis and in our analysis), on the indications for which SecOPs were offered, or on the method of provision. Most SHIs participating in our survey were also included in their study. We added information on two SHIs that were assessed to have no SecOPs by them and information on SecOPs by PHIs. Furthermore, we included information that may not have been provided on websites and investigated whether evaluations were performed. Beyond them, we are not aware of any analysis that is comparable to ours.

### Strengths and limitations

First, there are limitations regarding the way health insurers completed our questionnaire. One health insurer only answered the first part of our survey, arguing that the remaining parts refer to internal processes. It remains unclear whether some participants misunderstood our questions on the conduct of evaluations. We meant to include all evaluations (including, but not restricted to evaluations on costs only). Some parts of our survey (particularly the part on evaluations) were answered inconsistently. One example is the agreement between first and second opinions. Several health insurers answered this question even though they did not state that they have performed evaluations and/or measured the level of agreement. Furthermore, the majority of health insurers answered that they offer SecOPs for indications, interventions or treatments according to §27b SGB V. However, in the list of indications for which a SecOP was provided, most health insurers did not refer to the Second Opinion Directive. This is particularly true when considering the fact that our survey was performed before shoulder arthroscopy was added to the list of indications.

Another important limitation is the response rate of 19%, which should be considered when interpreting our results. Our method of contacting health insurers may have contributed to this low rate. Because of the heterogeneity in the responsibility and administration of the SecOPs, we contacted the central offices and asked them to forward our questionnaire to the responsible parties. To ensure that our questionnaire reached the responsible parties, we contacted each health insurer that had neither participated in our survey nor refused to participate a second time. Nonetheless, our response rate fits the expected response rate for postal questionnaires [20]. Our coverage of included health insured persons is higher (34 and 40% of people insured by PHIs and SHIs, respectively, excluding one anonymous health insurer) because larger health insurers tended to be included. On the one hand, the tendency to include larger health insurers is another restriction to the generalizability of our results. For example, larger health insurers offer SecOPs more often than smaller ones [19]. On the other hand, the tendency results in a higher coverage of health insured persons making it possible to provide results affecting a higher share of people. Another strength of our work is that all SHIs and PHIs in the country were contacted. Our results show that there are some discrepancies between SHIs and PHIs, leading to the necessity to add information about the SecOPs offered by PHIs. Furthermore, the development of a questionnaire that enables researchers to obtain information for SecOPs separately is a strength of our work. This is relevant when health insurers offer several SecOPs.

### Implications for research and practice

A follow-up survey of SecOPs provided by health insurers would help to examine the impact of the Second Opinion Directive in more detail. The SecOPs should be evaluated, and the evaluations should be published. This would enable the assessment of different methods for delivering second opinions (personal vs. submitted documents) and of different routes of delivery (outsourcing vs. selected health care providers vs. in house) as well as the comparison between different SecOPs in general. In particular, international comparisons between SecOPs would accelerate the process of advancing SecOPs and provide a broader understanding of the mechanisms, structures, advantages, and disadvantages of different SecOPs. Furthermore, reasons for the low number of participants in the SecOPs should be investigated.

## Conclusion

Overall, health insurers focused their SecOPs on orthopedics and oncology. Second opinions based on submitted documents only and personally delivered second opinions were equally weighted in the responses. Only half of the SecOPs were confirmed to have conducted a formal evaluation process or, if not, plan such a process in the future. Analysis grouped by type of insurer showed that there are some differences in the offered SecOPs such as the frequency of performing evaluations.

## Supplementary Information

**Additional file 1: ** Health insurers approach to SecOPs.

**Additional file 2:** Structure of SecOPs.

**Additional file 3:** Information about characteristics of participants in the SecOPs.

**Additional file 4:** Evaluations of SecOPs.

## Data Availability

The datasets used and/or analyzed during the current study are available from the corresponding author on reasonable request.
