# Supplementary material for: Analysis of second opinion programs provided by German statutory and private health insurance – a survey of statutory and private health insurers
Source: BMC Health Serv Res. 2021 Mar 9;21:209. doi: 10.1186/s12913-021-06207-8 (PMC7941885; doi:10.1186/s12913-021-06207-8)
Supplement: Supplementary file 1 — Additional file 1: Health insurers approach to SecOPs. [file 12913_2021_6207_MOESM1_ESM.docx]

Additional file 1. Health insurers approach to SecOPs.

|  | **Results for main analysis** | **Results for subgroup of statutory health insurers** | **Results for subgroup of private health insurers** |
| --- | --- | --- | --- |
| Do you offer second opinion programs for indications, interventions or treatments according to §27b SGB V? (%, n/A)^2^ | Yes (65%, 20/31)  No (29%, 9/31)  No (valid) answer (6%, 2/31) | Yes (63%, 15/24)  No (33%, 8/24)  No (valid) answer (4%, 1/24) | Yes (67%, 4/6)  No (17%, 1/6)  No (valid) answer (17%, 1/6) |
| Do you offer second opinion programs to indications, interventions or treatments beyond § 27b SGB V? (%, n/A)^2^ | Yes (90%, 28/31)  No (10%, 3/31) | Yes (88%, 21/24)  No (13%, 3/24) | Yes (100%, 6/6) |
| If currently no second opinion program is provided at all, did you offer such programs in the past? (%, n/B)^2^ | Yes (0%, 0/3)  No (100%, 3/3) | Yes (0%, 0/3)  No (100%, 3/3) | / |
| If currently no second opinion program is provided at all, do you plan to introduce such a program in the following 2 years? (%, n/B)^2^ | Yes (0%, 0/3)  No (67%, 2/3)  No (valid) answer (33%, 1/3) | Yes (0%, 0/3)  No (67%, 2/3)  No (valid) answer (33%, 1/3) | / |
| For which indications, interventions, or treatments do you provide second opinion programs? (%, n/C)^1^ | Orthopedics (75%, 21/28)  Oncology (71%, 20/28)  Cardiology (11%, 3/28)  Gynecology (11%, 3/28)  Pediatrics (11%, 3/28)  Urology (11%, 3/28)  Dermatology (7%, 2/28)  Ear, nose, and throat (7%, 2/28)  Gastroenterology (7%, 2/28)  General and Visceral Surgery (7%, 2/28)  Ophthalmology (7%, 2/28)  Other (18%, 5/28)  No restriction (21%, 6/28) | Orthopedics (81%, 17/21)  Oncology (67%, 14/21)  Gynecology (5%, 1/21)  Pediatrics (5%, 1/21)  Urology (5%, 1/21)  Other (14%, 3/21)  No restriction (14%, 3/21) | Oncology (83%, 5/6)  Cardiology (50%, 3/6)  Orthopedics (50%, 3/6) Dermatology (33%, 2/6)  Ear, nose, and throat (33%, 2/6)  Gastroenterology (33%, 2/6)  General and Visceral Surgery (33%, 2/6)  Gynecology (33%, 2/6)  Ophthalmology (33%, 2/6)  Pediatrics (33%, 2/6)  Urology (33%, 2/6)  Other (33%, 2/6)  No restriction (50%, 3/6) |
| According to which criteria do you select the included indications? (%, n/C)^1^ | Potential impact on a patients (79%, 22/28)  Insured persons’ demand (68%, 19/28)  Economic importance (61%, 17/28)  Indication quality (50%, 14/28)  Oversupply (43%, 12/28)  Compliance with guidelines (25%, 7/28)  Number of potential participants (25%, 7/28)  Second Opinion Directive (21%, 6/28)  Other criteria (4%, 1/28)  No (Valid) answer (4%, 1/28) | Potential impact relevant to patients (71%, 15/21)  Insured persons’ demand (57%, 12/21)  Economic importance (52%, 11/21)  Indication quality (52%, 11/21)  Oversupply (38%, 8/21)  Second Opinion Directive (29%, 6/21)  Number of potential participants (24%, 5/21)  Compliance with guidelines (19%, 4/21)  Other criteria (5%, 1/21)  No (valid) answer (5%, 1/21) | Insured persons’ demand (100%, 6/6)  Potential impact relevant to patients (100%, 6/6)  Economic importance (83%, 5/6)  Oversupply (67%, 4/6)  Compliance with guidelines (50%, 3/6)  Indication quality (33%, 2/6)  Number of potential participants (33%, 2/6) |

*^1^ multiple answers possible*

*^2^ multiple answers NOT possible*

*A number of health insurers participating*

*B number of health insurers without SecOPs*

*C number of health insurers with SecOPs*
