# Supplementary material for: Analysis of second opinion programs provided by German statutory and private health insurance – a survey of statutory and private health insurers
Source: BMC Health Serv Res. 2021 Mar 9;21:209. doi: 10.1186/s12913-021-06207-8 (PMC7941885; doi:10.1186/s12913-021-06207-8)
Supplement: Supplementary file 2 — Additional file 2: Structure of SecOPs. [file 12913_2021_6207_MOESM2_ESM.docx]

Additional file 2. Structure of SecOPs.

|  | **Results for main analysis** | **Results for subgroup of statutory health insurers** | **Results for subgroup of private health insurers** |
| --- | --- | --- | --- |
| Which further preconditions do the insured persons have to comply with for participation in the second opinion program? (%, n/D)^1^ | Referral to a hospital (27%, 12/44)  Doctor’s letter with indication for surgery (23%, 10/44)  Referral to second opinion (16%, 7/44)  No further requirements (20%, 9/44)  Other (59%, 26/44) | Referral to a hospital (34%, 11/32)  Doctor’s letter with indication for surgery (28%, 9/32)  No further requirements (22%, 7/32)  Referral to second opinion (22%, 7/32)  Other (50%, 16/32) | No further requirements (20%, 2/10)  Referral to a hospital (10%, 1/10)  Doctor’s letter with indication for surgery (10%, 1/10)  Other (80%, 8/10), e.g. existence of a comprehensive health insurance |
| Is participation in the second opinion program free of charge to the insured person? (%, n/D)^2^ | Yes (95%, 42/44)  No (2%, 1/44)  No (valid) answer (2%, 1/44) | Yes (94%, 30/32)  No (3%, 1/32)  No (valid) answer (3%, 1/32) | Yes (100%, 10/10)  No (0%, 0/10) |
| If participation in second opinion program is free of charge, how does accounting proceed? (%, n/E)^2^ | Direct payment by insurer (98%, 41/42)  Advance payment by insured person (2%, 1/42) | Direct payment by insurer (100%, 30/30) | Direct payment by insurer (90%, 9/10)  Advance payment by insured person (10%, 1/10) |
| Are expenses for travelling to the second opinion provider repaid by the insurer? (%, n/D)^2^ | Yes (7%, 3/44)  No (80%, 35/44)  No (valid) answer (14%, 6/44) | Yes (3%, 1/32)  No (84%, 27/32)  No (valid) answer (13%, 4/32) | Yes (20%, 2/10)  No (60%, 6/10)  No (valid) answer (20%, 2/10) |
| Which qualification criterion account for the second opinion provider? (%, n/D)^1^ | Expertise (68%, 30/44)  Exchange of experts (25%, 11/44)  Direct reference to Second Opinion Directive (14%, 6/44)  Neutrality/independence (14%, 6/44)  Organizational factors (11%, 5/44)  Criteria related to quality management (9%, 4/44)  Other (11%, 5/44) | Expertise (59%, 19/32) Direct reference to Second Opinion Directive (19%, 6/32)  Exchange of experts (19%, 6/32)  Neutrality/Independence (9%, 3/32)  Criteria related to quality management (9%, 3/32)  Organizational factors (6%, 2/32)  Other (16%, 5/32) | Expertise (90%, 9/10)  Exchange of experts (50%, 5/10)  Organizational factors (30%, 3/10)  Neutrality/Independence (10%, 1/10)  Criteria related to quality management (10%, 1/10) |
| How is independence of the second opinion from financial influencing factors ensured? (%, n/D)^1^ | Prohibiting second opinion providers from conducting the subsequent procedure (66%, 29/44)  Prohibiting the second opinion provider to work for the same company as the first opinion provider (57%, 25/44)  Other (20%, 9/44) | Prohibiting the second opinion provider to perform the subsequent treatment (66%, 21/32)  Prohibiting the second opinion provider to work for the same company as the first opinion provider (59%, 19/32)  Other (16%, 5/32) | Prohibiting the second opinion provider to perform the subsequent treatment (70%, 7/10)  Prohibiting the second opinion provider to work for the same company as the first opinion provider (60%, 6/10)  Other (30%, 3/10) |
| How do you inform insured persons about the second opinion programs provided? (%, n/D)^1^ | Internet (95%, 42/44)  Customer magazine (75%, 33/44)  Personal contact via insurance agents (45%, 20/44)  Information brochure (36%, 16/44)  Terms and conditions (7%, 3/44)  Other (36%, 16/44) | Internet (94%, 30/32)  Customer magazine (84%, 27/32)  Personal contact via insurance agents (44%, 14/32)  Information brochure (31%, 10/32)  Terms and conditions (6%, 2/32)  Other (31%, 10/32) | Internet (100%, 10/10)  Customer magazine (60%, 6/10)  Information brochure (60%, 6/10)  Personal contact via insurance agents (60%, 6/10)  Terms and conditions (10%, 1/10)  Other (40%, 4/10) |
| How are the second opinions delivered? (%, n/D)^2^ | Outsourcing to an external service provider (45%, 20/44)  Selected health service provider contracted with the health insurer (32%, 14/44)  Other (9%, 4/44), e.g. specific university hospitals  No (valid) answer (14%, 6/44) | Selected health service provider contracted with the health insurer (44%, 14/32)  Outsourcing to an external service provider (38%, 12/32)  Other (9%, 3/32)  No (valid) answer (9%, 3/32) | Outsourcing to an external service provider (60%, 6/10) Other (10%, 1/10)  No (valid) answer (30%, 3/10) |
| What is the basis for the provision of second opinions? (%; n/D)^2^ | Submitted documents only (48%, 21/44)  Direct contact between patient and doctor (45%, 20/44)  Contact by phone (2%, 1/44)  No (valid) answer (5%, 2/44) | Direct contact between patient and doctor (50%, 16/32)  Submitted documents only (41%, 13/32)  Contact by phone (3%, 1/32)  No (valid) answer (6%, 2/32) | Submitted documents only (60%, 6/10)  Direct contact between patient and doctor (40%, 4/10) |
| How are the second opinions communicated to the insured person? (%; n/D)^2^ | Both in written form and verbally (77%, 34/44)  Verbally only (11%, 5/44)  Written form only (11%, 5/44) | Both in written form and verbally (72%, 23/32)  Verbally only (16%, 5/32)  Written form only (13%, 4/32) | Both in written form and verbally (90%, 9/10)  Written form only (10%, 1/10) |
| Do you have timelines for the second opinion provision included in the contract with the second opinion provider? (%; n/D)^2^ | Yes (52%, 23/44)  No (45%, 20/44)  No (valid) answer (2%, 1/44) | Yes (50%, 16/32)  No (47%, 15/32)  No (valid) answer (3%, 1/32) | Yes (50%, 5/10)  No (50%, 5/10) |
| Do other health insurers offer the same second opinion program via the same service provider? (%; n/D)^2^ | Yes 66%, 29/44  No 32%, 14/44  No (valid) answer 2%, 1/44 | Yes 63%, 20/32  No 38%, 12/32 | Yes 70%, 7/10  No 20%, 2/10  No (valid) answer 10%, 1/10 |

*^1^ multiple answers possible*

*^2^ multiple answers NOT possible*

*D number of SecOPs*

*E number of SecOPs where participation is free of charge to the insured persons*
