# Supplementary material for: Analysis of second opinion programs provided by German statutory and private health insurance – a survey of statutory and private health insurers
Source: BMC Health Serv Res. 2021 Mar 9;21:209. doi: 10.1186/s12913-021-06207-8 (PMC7941885; doi:10.1186/s12913-021-06207-8)
Supplement: Supplementary file 3 — Additional file 3: Information about characteristics of participants in the SecOPs. [file 12913_2021_6207_MOESM3_ESM.docx]

Additional file 3. Information about characteristics of participants in the SecOPs

|  | **Results for main analysis** | **Results for subgroup of statutory health insurers** | **Results for subgroup of private health insurers** |
| --- | --- | --- | --- |
| How many insured persons participate in the second opinion program per year? median, interquartile range (based on X answers) | 31, 7-85 (26) | 30, 6-83 (20) | 36, 17-77 (6) |
| On average, how old are the participants? median, interquartile range (based on X answers) | 58 years, 56-60 (23) | 57 years, 54-60 (19) | 67 years, 64-68 (4) |
| What is the gender distribution of participants (% females) in the second opinion program? median, interquartile range (based on X answers) | 50%, 40-54% (25) | 50%, 43-54% (21) | 32%, 31-39% (4) |
| On average, how long does it take until the insured person gets the second opinion? median, interquartile range (based on X answers) | 9 days, 5-15 (23) | 8 days, 5-17 (18) | 10 days, 6-12 (5) |
