# Supplementary material for: Analysis of second opinion programs provided by German statutory and private health insurance – a survey of statutory and private health insurers
Source: BMC Health Serv Res. 2021 Mar 9;21:209. doi: 10.1186/s12913-021-06207-8 (PMC7941885; doi:10.1186/s12913-021-06207-8)
Supplement: Supplementary file 4 — Additional file 4: Evaluations of SecOPs. [file 12913_2021_6207_MOESM4_ESM.docx]

Additional file 4. Evaluations of SecOPs.

|  | **Results for main analysis** | **Results for subgroup of statutory health insurers** | **Results for subgroup of private health insurers** |
| --- | --- | --- | --- |
| Do you verify the second opinion’s indication quality? (%, n/D)^2^ | Yes (36%, 16/44 )  No (48%, 21/44) No (valid) answer (16%, 7/44) | Yes (28%, 9/32)  No (59%, 19/32)  No (valid) answer (13%, 4/32) | Yes (70%, 7/10)  No (10%, 1/10)  No (valid) answer (20%, 2/10) |
| Do you evaluate the SecOPs or did you evaluate them in the past? (%, n/D)^2^ | Yes (27%, 12/44)  No (50%, 22/44)  No (valid) answer (23%, 10/44) | Yes (19%, 6/32)  No (66%, 21/32)  No (valid) answer (16%, 5/32) | Yes (60%, 6/10)  No (10%, 1/10)  No (valid) answer (30%, 3/10) |
| If no evaluations are/were done, do you plan evaluations in the following 2 years? (%, n/F)^2^ | Yes (45%, 10/22)  No (45%, 10/22)  No (valid) answer (9%, 2/22) | Yes (43%, 9/21)  No (48%, 10/21)  No (valid) answer (10%, 2/21) | Yes (100, 1/1)  No (0%, 0/1) |
| If evaluations are/were done, what was measured during the evaluations? (%, n/G)^1^ | Satisfaction (75%, 9/12)  Agreement between first and second opinion (67%, 8/12)  Costs (58%, 7/12)  Second opinion’s quality (50%, 6/12)  Other (42%, 5/12) | Agreement between first and second opinion (100%, 6/6)  Costs (67%, 4/6)  Satisfaction (67%, 4/6)  Second opinion’s quality (17%, 1/6)  Other (67%, 4/6) | Satisfaction (83%, 5/6)  Second opinion’s quality (83%, 5/6)  Costs (50%, 3/6)  Agreement between first and second opinion (33%, 2/6)  Other (17%, 1/6) |
| If evaluations were done and agreement was measured, what was the level of agreement? 1(no agreement) to 5 (very high agreement) (%, n/H)^2^ | 1 (0%, 0/8)  2 (38%, 3/8)  3 (25%, 2/8)  4 (38%, 3/8)  5 (0%, 0/8) | 1 (0%, 0/6)  2 (33%, 2/6)  3 (17%, 1/6)  4 (50%, 3/6)  5 (0%, 0/6) | 1 (0%, 0/2)  2 (50%, 1/2)  3 (50%, 1/2)  4 (0%, 0/2)  5 (0%, 0/2) |
| All answer included: what was the level of agreement? 1(no agreement) to 5 (very high agreement) (%, n/I)^2^ | 1 (0%, 0/17)  2 (59%, 10/17)  3 (24%, 4/17)  4 (18%, 3/17)  5 (0%, 0/17) | 1 (0%, 0/13)  2 (62%, 8/13)  3 (15%, 2/13)  4 (23%, 3/13)  5 (0%, 0/13) | 1 (0%, 0/4)  2 (50%, 2/4)  3 (50%, 2/4)  4 (0%, 0/4)  5 (0%, 0/4) |
| If evaluations were done, does a report exist? (%, n/G)^2^ | Yes (67%, 8/12)  No (25%, 3/12)  No (valid) answer (8%, 1/12) | Yes (100%, 6/6)  No (0%, 0/6) | Yes (33%, 2/6)  No (50%, 3/6)  No (valid) answer (17%, 1/6) |
| If a report exists, would you make us the report available? (%, n/J)^2^ | Yes (25%, 2/8)  No (63%, 5/8)  No (valid) answer (13%, 1/8) | Yes (17%, 1/6)  No (67%, 4/6)  No (valid) answer (17%, 1/6) | Yes (50%, 1/2)  No (50%, 1/2) |

*^1^ multiple answers possible*

*^2^ multiple answers NOT possible*

*D number of SecOPs*

*F Number of SecOPs without evaluations*

*G Number of SecOPs with evaluations*

*H Number of SecOPs with evaluations and measurement of level of agreement between first and second opinion*

*I number of SecOPs for which an answer was provided regarding the level of agreement between first and second opinion (irrespective of the previous answers)*

*J number of SecOPs with evaluations performed and report existing*
